# The Role of Location-Context Binding in Nonspatial Visual Working Memory

**DOI:** 10.1523/ENEURO.0430-20.2020

**Published:** 2020-12-15

**Authors:** Ying Cai, Jacqueline M. Fulvio, Qing Yu, Andrew D. Sheldon, Bradley R. Postle

**Affiliations:** 1 Department of Psychology and Behavioral Science, Zhejiang University, Hangzhou 310007, China; 2 Department of Psychology, University of Wisconsin–Madison, Madison, WI 53706; 3 Medical Scientist Training Program and Neuroscience Training Program, University of Wisconsin–Madison, Madison WI 53706; 4 Department of Psychiatry, University of Wisconsin–Madison, Madison, WI 53706

**Keywords:** fMRI

## Abstract

Successful retrieval of an item from visual working memory (VWM) often requires an associated representation of the trial-unique context in which that item was presented. In experiment 1, fMRI of 16 male and female humans replicated a previous dissociation of the effects of manipulating memory load in comparison to the effects of manipulating context binding, by comparing VWM for one oriented line versus for three lines individuated by their location versus for three “heterogeneous” items drawn from different categories (orientation, color, and luminance): delay-period fMRI signal in frontal cortex and intraparietal sulcus (IPS) was sensitive to stimulus homogeneity rather than to memory load per se. Additionally, inspection of behavioral performance revealed a broad range of individual differences in the probability of responses to nontargets (also known as “swap errors”), and a *post hoc* comparison of high swap-error versus low swap-error groups generated several intriguing results: at recall, high swap-error subjects were seen to represent both the orientation and the location of the probed item less strongly, and with less differentiation from nonprobed items, and delay-period signal in IPS predicted behavioral and neural correlates of context binding at recall. In experiment 2, which was a preregistered replication, the 27 male and female humans were grouped into low and high swap-error groups by median split, and the results were broadly consistent with experiment 1. These results present a neural correlate of swap errors, and suggest that delay-period activity of the IPS may be more important for the operation of context binding than for representation per se of stimulus identity.

## Significance Statement

Although we often think of the contents of visual working memory (VWM) as representations of the items that need to be remembered, each item’s trial-unique context is also critical for successful performance. For example, if one observes a red, then a black, then a blue car passing through an intersection, vivid memory for the colors, alone, would not allow one to execute the instruction “follow the first of the three cars that just drove by.” Although manipulating load is commonly assumed to isolate storage functions, requiring memory for multiple items drawn from the same category also increases demands on the context binding needed to individuate these items. This experiment tracked the influence of context binding on VWM stimulus processing.

## Introduction

Individual differences in human visual working memory (VWM) capacity result from several factors, including the strategic deployment of attention (Fukuda et al., 2015), retrieval-related processes (Unsworth et al., 2014) and resistance to interference (Vogel et al., 2005). In fMRI studies, delay-period activity in the intraparietal sulcus (IPS) scales with the number of items in the memory set before asymptoting at an individual’s VWM capacity (Todd and Marois, 2004, 2005), and has often been assumed to reflect stimulus representation in VWM (Bettencourt and Xu, 2016; Xu, 2017). Here, we test the possibility that this activity may also reflect context binding: the association of each item in the memory set with its unique episodic context. In tests that require VWM for an array of colored squares, for example, the subject must remember not only each of the colors, but also the location at which each color was presented. Intact memory for all the colors but an impaired representation of which had been presented where can lead to “swap errors” (Schneegans and Bays, 2017). Indeed, some theoretical accounts hold that context binding is the factor that determines whether a stimulus can meaningfully be said to be “in” VWM (Oberauer and Lin, 2017).

In one previous study (Gosseries et al., 2018), we sought to unconfound the storage demands of a high-load condition from its context-binding demands by varying stimulus category homogeneity within the memory set: subjects were asked to remember either the direction of motion in one random-dot kinematogram (RDK; 1 M trials), the directions in three RDKs (3 M), or the direction in one RDK plus the colors of two color patches (1M2C). Although delay-period activity of the IPS was elevated during 3 M relative to 1 M trials, it was comparable for 1M2C and 1 M trials, indicating sensitivity to a factor other than memory load per se. In this experiment, the critical context was ordinal position, because stimuli on three-item trials were presented serially and all in the same location, with the item to be recalled prompted by a digit indicating “first,” “second,” or “third.”

In the present experiment, we tested context binding in VWM more directly. First, we changed the behavioral task such that stimulus location was the critical contextual dimension. Because the neural correlates of spatial processing are better understood than those of ordinal processing, this allowed a more direct assessment of variation in the representation of stimulus context. Second, we measured individual differences in swap error rate, a behavioral index of context binding, to relate variability in context binding efficacy with neural signals.

In a preliminary fMRI study enrolling 16 healthy young adults (experiment 1), we replicated the earlier finding that aggregated delay-period activity in IPS is more sensitive to category homogeneity in the stimulus set than to the number of items per se, reinforcing the association of this region with functions other than stimulus representation. Additionally, *post hoc* categorization of subject by swap error rate revealed several intriguing differences in the neural representation of stimulus content (orientation) and context (location). These gave rise to a set of preregistered hypotheses that were tested in experiment 2. We believed that the results from the preregistered experiment (presented here as experiment 2) would provide novel insight into the interpretation of delay-period signals, and the computations underlying context-binding in VWM.

## Materials and Methods

### Preregistered hypotheses

In this and all subsequent sections of this manuscript, we will make the distinction between “experiment 1,” the preliminary dataset and results from which our predictions arose, and “experiment 2,” for which we preregistered the a priori set of hypotheses listed in this section, and the methods that we planned to use to generate and analyze *de novo* data to test these hypotheses. Importantly, whereas two of the hypothesis generating results from experiment 1 can be characterized as “*post hoc* dichotomization of participants based on a median split on scores of some variable of interest” (Yarkoni and Braver, 2010), which can be inferentially problematic, an a priori extreme groups design can be an effective way to “increase […] power to detect effects by reducing the variance between participant groups relative to the variance within groups, thereby inflating effect sizes and making them easier to detect” (Yarkoni and Braver, 2010). (Note that for studies with preregistered hypotheses, the convention is to state these hypotheses in the Introduction or the very beginning of Materials and Methods; as a consequence, explanation of the rationale behind specific predictions, and of the names of some of the measures in which the hypotheses are framed, appear in a subsection at the end of Materials and Methods, Implementation of a priori hypothesis tests.)

#### Hypothesis 1 (extreme-group analysis)

The orientation recall specificity effect will be significantly greater, at TR10, for the low swap-error group than for the high swap-error group.

#### Hypothesis 2 (extreme-group analysis)

The location recall specificity effect will be significantly greater, at TR10, for the low swap-error group than for the high swap-error group.

#### Hypothesis 3 (continuous analysis conducted with Spearman correlation of rank ordering)

Individual differences in delay-period representation of stimulus locations, as assessed with multivariate inverted encoding modeling (IEM), will correlate with individual differences in swap-error rate (i.e., the “probability of a nontarget response,” or *p*N, parameter, to be described below, Analysis of behavioral data). (Note that the choice of a Spearman correlation reflects the fact that subjects in the low swap-error group will necessarily have *p*N values close to 0.)

#### Hypothesis 4 (continuous analysis conducted with Pearson correlation)

Individual differences in delay-period representation of stimulus location, operationalized as the amplitude of the IEM reconstruction of the probed item, will correlate with individual differences in the location recall specificity effect.

Precise details about how each of these hypotheses was generated and tested, and how the *n* needed for adequate statistical power was calculated, are presented in the final subsection below, Implementation of a priori hypothesis tests.

#### Additional analyses and “secondary hypotheses”

In addition to the theoretically important hypotheses listed above, we planned to carry out several additional analyses that can be considered of “secondary” importance for experiment 2, because their outcomes will have little-to-no consequence for our theoretically motivated predictions. Some of these entailed replication of analyses of BOLD signal-intensity data from experiment 1, that were, themselves, replications of previous findings. Others can be thought of as being “descriptive,” although they may literally entail carrying out a significance test. In this latter category, for example, it is important to determine the TRs at which the IEM reconstruction of targeted stimulus orientation is statistically reliable, for both the 1O and the 3O conditions. Furthermore, it is of interest (but not critical for hypotheses 1–4) to know whether any reliable reconstructions for the low swap-error group differ quantitatively from those of the high swap-error group. These will be identified as secondary hypotheses.

### Subjects

For experiment 1, 18 right-handed volunteers [10 females, aged 18–25 years; mean (SD) = 21.70 (1.75)] from the University of Wisconsin–Madison community participated in a behavior-only experiment for remuneration ($10/h). Two of the 18 elected not to participate in the subsequent fMRI study, and remaining 16 subjects [eight females, aged 18–25 years; mean (SD) = 20.50 (1.78)] participated the fMRI experiment for additional remuneration ($20/h). All subjects provided informed consent according to the procedures approved by the Health Sciences Institutional Review Board at the University of Wisconsin–Madison. Subjects had normal or corrected-to-normal vision, no reported history of neurologic or psychiatric disease, and, for the fMRI experiment, no contraindications for MRI.

For experiment 2, we conducted an extreme-group analysis in which the initial step was to recruit healthy adult volunteers within the age range of 18–25 years, from the University of Wisconsin–Madison community, for a behavioral screening session comprising 100 trials of delayed recall (also known as “delayed estimation”) of orientation with a set size of three. Stimuli and procedures closely followed those from 3O trials from experiment 1 (see below, Stimuli). The resultant recall data were fit with the three-factor mixture model described below (Behavioral tasks), and subjects with swap error rates of <5% were selected for the fMRI sessions as part of the low swap-error group, and subjects with swap error rates >12% were selected as part of the high swap-error group. The preregistered plan called for between 11 and 18 subjects for the low swap-error group, and between 11 and 18 subjects for the high swap-error group. The *N*s required for each group were planned as ranges, to reflect the following factors (detailed below, Implementation of a priori hypothesis tests): (1) power analysis indicated that 29 subjects were required to achieve 90% power to detect the effect predicted by hypothesis 3, but because this was a continuous analysis, it did not constrain how many of these 29 needed to belong to either extreme group; and (2) power analysis for a secondary hypothesis indicated that 11 subjects were needed to achieve 90% power to observe a significant reconstruction of stimulus orientation at TR10, in occipital cortex, on 3O trials. Because all other a priori estimated *N*s were lower than these numbers, our stopping point for screening was when we had successfully recruited and tested a minimum of 11 subjects with swap error rates of <5%, a minimum of 11 subjects with swap error rates of >12% (selected to ensure reasonable balance between the two groups), and a total of 29 subjects. (All subjects were paid at the end of the behavioral testing session and departed from the laboratory before their data were analyzed. They were told that they might be subsequently recontacted to be invited to participate in an fMRI study. Those re-contacted for the fMRI sessions of experiment 2 were not informed of how they had been categorized with regard to *p*N, nor were they informed about the concept or definition of a swap error. It was also possible that individuals whose *p*N excluded them from experiment 2 could be recontacted to participate in a different study being conducted by our research group.)

Note that, because it is measures of *p*N acquired during the fMRI session that we used to test our hypotheses, there were two possible developments that could have required us to recruit and behaviorally screen more individuals than the number listed above. The first was if the number of subjects whose *p*N from the fMRI scanning session satisfying the criterion for the low swap-error group dropped below 11, and the second was if any subject’s fMRI data were unusable, whether because of withdrawal from the study or poor data quality.

### Stimuli

In experiment 1 there were three trial types: delayed recall (also known as “delayed estimation”) of an oriented bar (1O), of one from a memory set of three oriented bars (3O), or of one item from a memory set of 1 oriented bar, 1 color patch, and 1 luminance patch (1O1C1L). Oriented-bar stimuli (length, 4°; width, 0.08°) were rendered as the black diameter of a white circular patch. Sample stimuli could appear in one of nine possible orientations ranging from 0° to 160°, in 20° increments, with a jitter of ±1–5° determined randomly on each trial. Color stimuli were presented on 4°-diameter circles, and drawn from a pool of 9 colors that were equidistant along a circle in CIE L*a*b* color space (L = 70, a = 20, b = 38, radius of 60; sample items were therefore equiluminant, varying mainly in hue and slightly in saturation), with a randomized jitter of ±1–5° on each trial. Luminance stimuli comprised a gray annulus (diameter = 2.67°) inside a white ring (RGB values ([0, 0, 0]; diameter = 4°). The annulus could take on one of nine grayscale values ranging equidistantly from light gray [0.03, 0.03, 0.03] to darkest gray [0.97, 0.97, 0.97], with jitter of ±1–5° determined randomly on each trial.

On all trials, masks were rendered as a white circular patch bisected by 18 black 0.08° × 4° bars, all intersecting at their midpoints and each separated in orientation by 10°.

**Figure 1.**
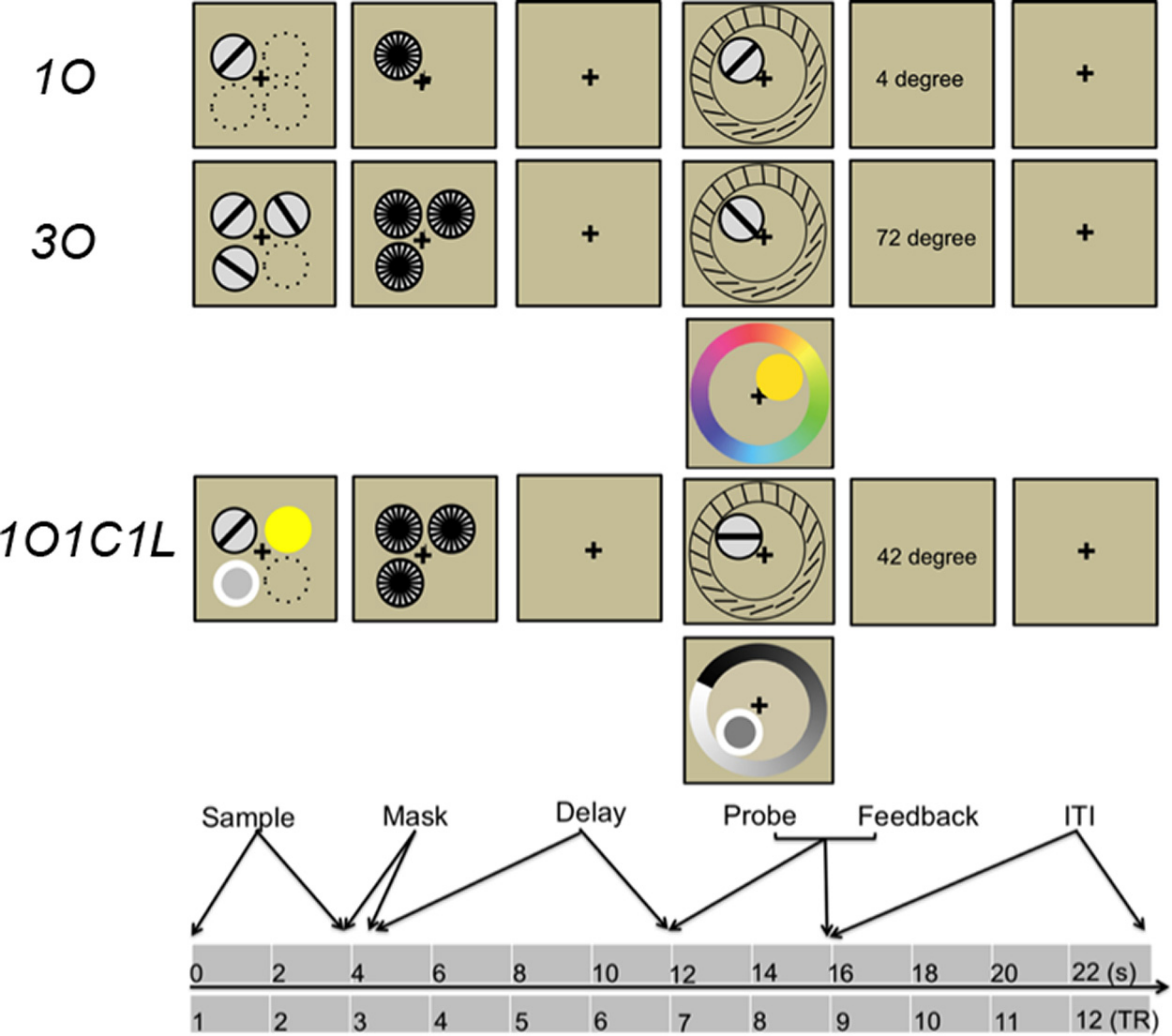
Schematic illustration of the three trial types from experiment 1 (1O = one orientation; 3O = three orientations; 1O1C1L = one orientation, one color, one luminance). Dotted circles indicate the other possible stimulus presentation locations, but were not presented during experiment. Experiment 2 did not include 1O1C1L trials.

Recall displays comprised a circular stimulus patch, initially “empty,” and a response wheel centered on fixation, with a radius to its outer edge of 9.2° and a width of 2°. Varying continuously around the response wheel were all possible values of the category being tested. For orientation, this was rendered as 20 equally spaced black bars (0.05° × 1.8°), ranging in orientation from 0° to 171°, in 9° increments. For color and luminance, all 180 values of that dimension were evenly distributed along the circle. The angle of rotation of the response wheel varied unpredictably from trial-to-trial, to discourage response planning during the delay. At the onset of the recall display a cursor (a conventional “mouse” arrow) was always positioned at central fixation, and the stimulus patch was rendered with a randomly determined value rendered in the format of the sample stimuli. As soon as the subject began to move the trackball of the response box (see Behavioral tasks) the cursor moved correspondingly, and the stimulus patch took on the value corresponding to the location on the response wheel that was nearest to the cursor. Throughout the experiment, the background screen color was gray [0.5, 0.5, 0.5] (Fig. 1).

Experiment 2 included only 1O and 3O trial types (for rationale, see below, Behavioral task during fMRI scanning), and the stimuli differed from experiment 1 in that sample stimuli appeared in one of six possible locations, with orientations ranging from 0° to 150°, in 30° increments, with a jitter of ±1–5° determined randomly on each trial.

### Behavioral task

In experiment 1, each trial of the 1O task began with the 4 s presentation of a sample item equiprobably and unpredictably at one of four possible locations, each in one quadrant of the screen, and each with horizontal and vertical eccentricities from fixation of 5°. The identity of the sample, drawn equiprobably and unpredictably from a pool of nine orientations, varied independently of location. The 8-s delay period began with a mask presented at the same location as the sample. Responses were made by moving a cursor with a trackball and “clicking” on the recalled orientation with a button press. As soon as the trackball began to move, a bar appeared within the circular patch with an orientation, updating in real-time, that matched the orientation on the wheel that was closest to the cursor. RT was computed as the latency between response-wheel movement onset and button press. Feedback, indicating the error between the recalled orientation and the sample orientation (in degrees) was presented centrally, replacing the fixation cross, appearing immediately after the response until the end of the 4-s response window. Intertrial interval (ITI) was 2 s for the behavior-only experiment, and 8 s for the fMRI experiment. A black fixation cross was present at the center of the screen throughout each block of trials, and subjects were instructed to fixate it throughout the block.

3O trials followed the same procedure was as 1O, with the following adjustments necessitated by the greater number of items: on each trial, each of the three sample stimuli was drawn randomly from the pool of nine, without replacement; the three were displayed simultaneously, with each of the four possible configurations of location occurring equiprobably and unpredictably; each of the three stimulus locations was backward masked; and the location of the probed item occurred equiprobably and unpredictably. 1O1C1L followed the same procedure was as for 3O, except that each trial featured one sample item drawn from each of the three stimulus categories, and the category of the probed item occurred equiprobably and unpredictably (Fig. 1).

In experiment 2, the task procedure was the same as that in experiment 1, with two exceptions: only 1O and 3O trials were included, and stimuli could appear at six evenly spaced locations along the imaginary circumference of a circle 5° in diameter, centered on fixation.

#### Behavior-only experiment from experiment 1

Testing was broken into two blocks of 1O and 3O trials and three blocks of 1O1C1L trials. All blocks contained 50 trials, and block order was counterbalanced across subjects by drawing the first 18 orders from a Latin square. Each block of 1O and 3O trials presented 25 of each, in a randomized sequence (thereby yielding a total of 50 1O responses and 50 3O responses per subject), and each block of 1O1C1L trials included 17 probes of two of the categories and 16 of the remaining category, randomized within block and balanced across the three blocks, thereby yielding 50 1O1C1L orientation responses, 50 1O1C1L color responses, and 50 1O1C1L luminance responses. All the experimental stimuli were controlled by the Psychophysics Toolbox (http://psychtoolbox.org; Brainard, 1997) running in MATLAB (MathWorks), presented on a 60-Hz projector with a screen width of 32.5 cm (iMac). The viewing distance was 62 cm.

#### Behavioral screening for experiment 2

Procedures for the orientation WM task were identical to those from experiment 1, with the exception that subjects only performed two 50-trial blocks of the 3O task.

#### Behavioral task during fMRI scanning

For experiment 1, there were two scanning sessions, and during the first session subjects first performed four blocks of three-item trials: nine trials of 3O and nine trials 1O1C1L in a randomly determined order during each block; three probes of each category on 1O1C1L trials in a randomly determined order during each block. Next, subjects completed eight 18-trial blocks of 1O trials, with each orientation appearing twice in a randomly determined order during each block. In the second fMRI scanning session, subjects performed an additional 12 18-trial blocks of 1O trials. (The larger number of 1O trials was needed to train the IEM models with which data from all trial types would be tested.)

**Figure 2.**
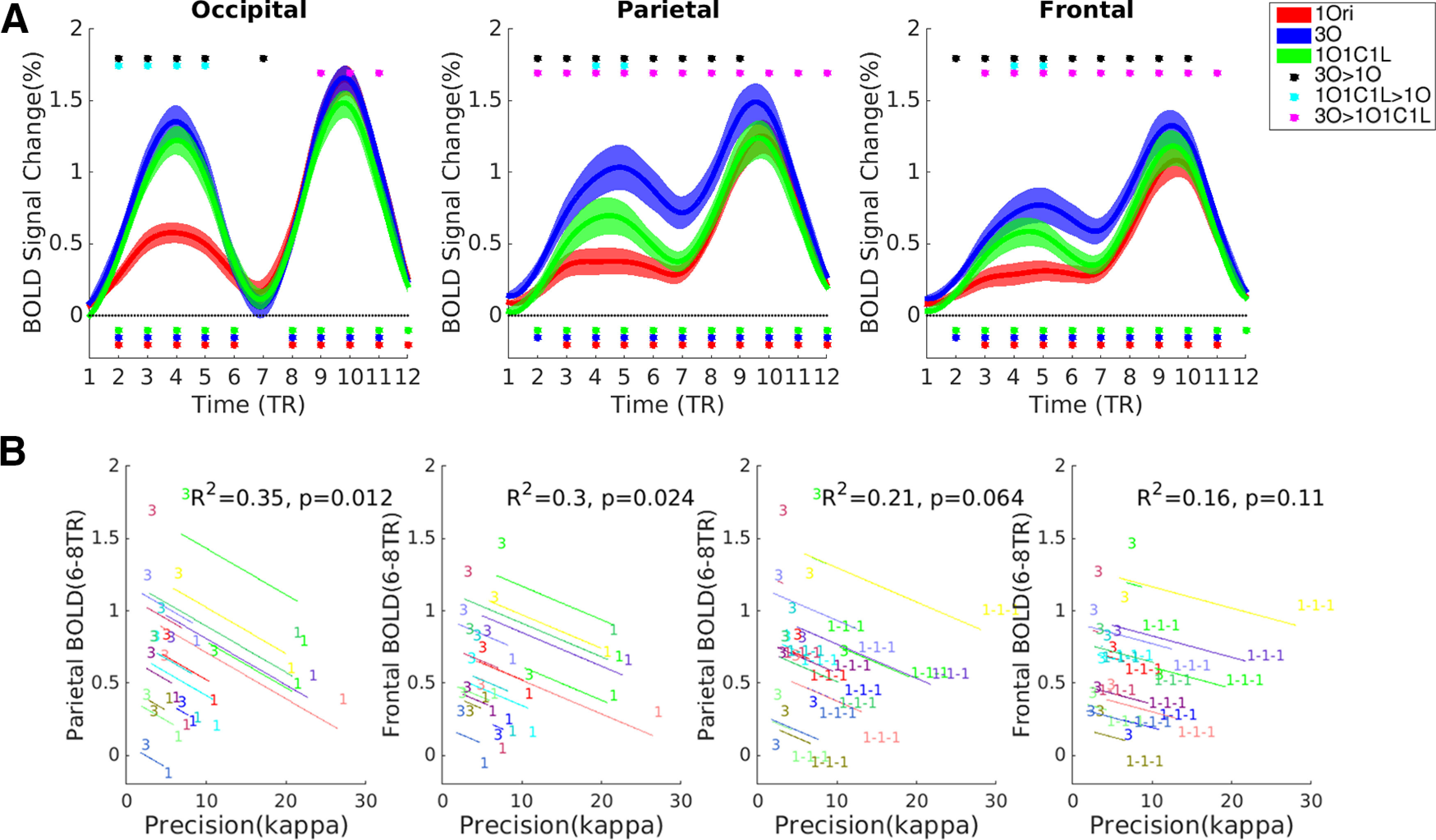
BOLD signal intensity results from three ROIs in experiment 1. ***A***, Trial-averaged BOLD signal. Dots below the *x*-axis indicate significance versus baseline; dots above the plots indicate significant differences between trial types. ***B***, Within-subject correlations (ANCOVAs) between delay-period BOLD signal intensity and behavioral precision of recall. In each plot, data from each subject are portrayed in a different color. The “1,” “3,” and “1-1-1” symbols indicate individual values in the 1O, 3O, and 1O1C1L tasks, respectively. Thus, for example, the left-most plot illustrates that on 3O trials, delay-period BOLD signal in IPS is high and precision is low, whereas on 1O trials, delay-period BOLD signal in IPS is low and precision is high. The slope of the lines indicates the tendency at the group level, and the offsets between lines indicate where different individuals sit within this space.

For experiment 2, subjects performed only 1O and 3O trials. The rationale was that the most novel effects observed in experiment 1 were the differences in IEM reconstruction of orientation between low versus high swap-error subjects. These differences were of greatest theoretical significance on 3O trials, which place the greatest demands on context binding. Furthermore, the results from experiment 1 involving comparison of patterns of BOLD signal intensity in the 1O versus 1O1C1L versus 3O conditions, and their relation to behavior (Fig. 2), were robust and adequately powered at *n* = 16, and were, themselves, replications of a previous result (Gosseries et al., 2018). Therefore, for the experiment 2, we maximized the sensitivity of within-subject IEM reconstructions, and of between-group comparisons, by administering 108 3O trials (an increase from the 36 that featured in experiment 1).

For the fMRI portion of experiment 2, each subject completed two 2-h scanning sessions, each on a separate day. The first scanning session began with six 18-trial blocks of 3O trials, followed by seven blocks of 1O trials. The second scanning session consisted of thirteen 18-trial blocks of 1O trials. Each block of trials was performed during a 7.2-min scan. Thus, each scanning session entailed 1 h 33 min 36 s of fMRI scanning, not including the brief pauses between scans. Over the course of all 108 3O trials, each orientation was probed at each location three times. Over the course of all 360 1O trials, each orientation was presented (and probed) at each location a total of ten times at each location.

#### Analysis of behavioral data

For experiment 1, reaction time (RT) of the response-ending button press was collected and raw response error distance estimated by the distance on the response wheel between the subjects’ selection and the true target value (in degrees). Trials without responses were excluded. For 1O and 1O1C1L trials, response error was fit to a two-factor mixture model that estimated the proportion of responses made to the sample (i.e., the probability of a target response (*p*T), and the probability of guess responses (*p*U), as well as the precision of target responses (κ; Bays et al., 2009). For 3O trials a third factor, the probability of a response to a nontarget (*p*N; also known as “swap error”), was included in the model. Parameter estimates were obtained using maximum-likelihood estimation (expectation maximization) using MATLAB routines available at http://www.bayslab.com. Differences across trial type in descriptive measures and in model parameters were assessed with repeated measures one-way ANOVAs, and significant effects were followed up with paired *t tests*. For descriptive measures, we only focused on response error, because the RT was necessarily noisy because it included the time to adjust the response dial with a trackball positioned adjacent to the thigh of the supine subject. For model estimates, we focused on κ and *p*T, because *p*U was highly collinear with *p*T in 1O and 1O1C1L trials (i.e., for the two-factor mixture model, *p*U+*p*T* *=* *1). Note that *p*N could only be estimated in 3O trials.

The *p*N parameter estimated from 3O trials provides a measure of the efficacy of context binding, because a swap error corresponds to a trial on which the subject has forgotten the location context of the item that they are recalling. For experiment 1, *post hoc* inspection of performance from the behavior-only experiment revealed a clear bimodal distribution in estimates of *p*N, with six subjects having a *p*N at or near 0 (indicating effectively no swap errors) and the remaining 10 subjects all having a *p*N of 0.127 or higher. Based on this pattern in the behavior-only experiment, subjects were grouped into low swap-error and high swap-error groups for the analyses of the fMRI experiment.

For the behavioral task in the fMRI component of experiment 1, RT and response errors were collected in the same way, but model fitting was only conducted for 1O trials because of the limited number of 3O and 1O1C1L trials. Correlations were conducted on all measures of behavioral performance on 1O trials from the two experiments, with the exception of RT, to assess the stability of subjects’ performance.

For experiment 2, procedures for analyses and model fitting of data from 1O and 3O trials were the same as those from experiment 1.

### fMRI methods

Unless specified otherwise, the fMRI methods for experiment 2 followed those from experiment 1, as described in this section.

#### General procedure and behavioral tasks

For experiment 1, the fMRI experiment comprised two scanning sessions, each lasting ∼1.5 h. The first of the two scanning sessions followed the behavior-only task by 6–21 d, and the second scanning session followed the first by 2–28 d. Scanning of three-item trials preceded scanning of 1O trials to minimize the likelihood that subjects would process orientation stimuli different from color and luminance stimuli on 1O1C1L trials. All the experimental stimuli were controlled by the Psychophysics Toolbox (http://psychtoolbox.org; Brainard, 1997) running in MATLAB (MathWorks), presented via a 60-Hz projector (Silent Vision 6011; Avotec) backprojecting onto a screen mounted inside the bore of the scanner, and viewed through a coil-mounted mirror. The viewing distance was 68.58 cm and screen width was 33.02 cm.

#### Data acquisition

Whole-brain images were acquired with a 3 Tesla scanner (Discovery MR750; GE Healthcare) at the Lane Neuroimaging Lab at the University of Wisconsin–Madison. For all subjects, a high-resolution T1-weighted image was acquired with a fast spoiled gradient-recalled-echo sequence (TR = 8.2 ms, TE =3.2 ms, Flip angle =12°, 160 axial slices, 256 × 256 in-plane, 1 mm isotropic). A T2*-weighted gradient echo pulse sequence was used to acquire data sensitive to the BOLD signal while subjects performed the VSTM task (TR = 2000 ms, TE = 25 ms, flip angle = 60°, within a 64 × 64 matrix, 42 sagittal slices, 3 mm isotropic). Each of the twenty fMRI scanning runs generated 213 volumes (excluding disdaqs).

#### Preprocessing

fMRI data were preprocessed using the Analysis of Functional Neuroimages (AFNI) software package (http://afni.nimh.nih.gov; Cox, 1996). All volumes were spatially aligned to the first volume of the first run using rigid-body realignment, then aligned to the T1 volume. Volumes were corrected for slice-time acquisition, and linear, quadratic, and cubic trends were removed from each run to reduce the influence of scanner drift. For univariate analyses, data were spatially smoothed with a 4-mm full-width at half-maximal Gaussian, and *z*-scored separately within run for each voxel. For IEM and MVPA analyses (see below), data were *z*-scored separately within run for each voxel, but were not smoothed. All analyses were conducted in each subject’s native space.

#### Data analysis

##### Univariate analyses and ROI creation

For experiment 1, a modified general linear model (GLM) was fit to data from all 3O and 1O1C1L trials and from 36 randomly selected 1O trials. It included regressors modeling the sample presentation, delay, and recall periods with boxcars of 4, 8, and 4 s, respectively, each convolved with the canonical hemodynamic response function supplied with AFNI.

A different set of anatomically constrained, functionally defined regions of interest (ROIs) was created for each of the three categories of analysis: BOLD signal intensity, IEM of stimulus orientation, and MVPA of stimulus location. For all three, anatomic regions were generated from the standard anatomic masks for occipital, parietal, and frontal cortex from the MNI152_T1_1mm template and warping them to each subject’s native space. For BOLD signal intensity analyses, an occipital sample ROI was generated for each subject by selecting the 400 voxels with the highest *t* values for the contrast [Sample_3O_ – Sample_1O_] within anatomically defined occipital cortex, and parietal delay and frontal delay ROIs were generated by selecting the 400 voxels with the highest *t* values for the contrast [Delay_3O_ – Delay_1O_] within each of these anatomically defined regions. Within each of these ROIs, trial-averaged time series for each of the three trial types were generated and converted to mean percentage signal change from baseline (first TR of the trial), and contrasts versus baseline and between conditions conducted with *t* tests (all *p* values false discovery rate (FDR)-corrected across TRs, ROIs, and comparisons). For IEM analyses, four sets of “sample location-specific” ROIs were generated with the top 400 voxels within each of the three anatomic regions responding to the contrasts [Sample_upper left_ – baseline], [Sample_upper right_ – baseline], [Sample_lower left_ – baseline], and [Sample_lower right_ – baseline]. Finally, for MVPA analyses, “location-general” ROIs were created with the top 400 voxels within each of the three anatomic regions identified with the contrast [(Sample_upper left_ + Sample_upper right_ + Sample_lower left_ + Sample_lower right_) – baseline]. (Analyses of fMRI data from 1O trials, unrelated to those described here, are presented in Cai et al. (2019)).

For experiment 2, ROI creation differed only in that it used data from all 3O trials and from 108 randomly selected 1O trials.

##### Task-related patterns of covariation

For experiment 1, we used ANCOVA to evaluate evidence for correlated sensitivity across trial types (specifically, across 1O vs 3O and across 1O1C1L vs 3O) of two of dependent variables, BOLD signal intensity and behavioral precision, seen to covary in previous studies (Emrich et al., 2013; Gosseries et al., 2018). Unlike simple correlations, ANCOVA accommodates the fact that each subject contributes a value for each level of the factor of trial type. It removes between-subject differences and assesses evidence for “within-subject correlation,” the extent to which variation in one dependent variable can be explained by variation in a second (Bland and Altman, 1995). For analyses including delay-period fMRI activity, the BOLD signal was averaged across TR6 and TR7, those least likely to be contaminated by sample-related signal.

For the experiment 2, comparable ANCOVAs, conducted with data from the 3O and 1O trials, were considered secondary hypotheses.

##### Multivariate IEM

In experiment 1, we estimated population-level neural representations of the orientation of oriented-bar stimuli with multivariate IEM (Serences and Saproo, 2012; Sprague et al., 2018). To optimize estimation from 1O trials, four IEMs were trained within each of the three anatomic ROIs, one for each location at which a sample could appear on the screen. The four resultant location-specific reconstructions were averaged before assessment of the results of IEM training and testing.

To build our IEMs we assumed that the responses of each voxel can be characterized by activity in 9 hypothesized tuning channels, one corresponding to each of the 9 possible sample orientations. Following previous work (Ester et al., 2015; Yu and Shim, 2017), the idealized feature tuning curve of each channel was defined as a half-wave-rectified and squared sinusoid raised to the seventh power. Before feeding the preprocessed data into the IEM, a baseline from each voxel’s response was removed in each run using the following equation from Brouwer and Heeger (2011):

$$B=B–m(m^{T}B),$$

in which *B* represented the data matrix from each run with size *v *×* c* (*v*: the 400 location-specific voxels; *c*: the nine orientations) and *m* represented the mean response across all stimulus conditions of length *v*. Next, for trials corresponding to each of the four sample locations, we randomly divided the data into a training set (81 trials) and a test set (nine trials; these numbers were selected to match the total number of 1O1C1L and 3O trials). We computed the weight matrix (*W*) that projects the hypothesized channel responses (*C_1_*) to actual measured fMRI signals in the training dataset (*B1*), and extracted the estimated channel responses (${\hat{C}}_{2}$) for the test dataset (*B2*) using this weight matrix. The relationship between the training dataset (*B1*, *v *×* n, n*: the number of repeated measurements) and the channel responses (*C_1_*, *k *×* n*) was characterized by:

$$B_{1}=WC_{1},$$

where *W* was the weight matrix (*v *×* k*).

Next, the least-squared estimate of the weight matrix ($\hat{W}$) was calculated using linear regression:

$$\hat{W}=B_{1}C_{1}^{T}{\left(C_{1}C_{1}^{T}\right)}^{-1}.$$

The channel responses (${\hat{C}}_{2}$) for the test dataset (*B2*) were then estimated using the weight matrix ($\hat{W}$):

$${\hat{C}}_{2}=\;{\left({\hat{W}}^{T}\hat{W}\right)}^{-1}{\hat{W}}^{T}B_{2}.$$

Having thus estimated the weight matrix mapping each voxel’s response to each orientation channel from the training dataset, we inverted this matrix to estimate channel responses on each test trial. The average response output for each channel across trials was obtained by circularly shifting each response to a common center of 0°. To generate smooth, 180-point channel tuning functions (CTF, also referred to as “reconstructions”) we repeated the encoding model analysis 180 times and shifted the centers of the orientation channels by 1° on each iteration (Brouwer and Heeger, 2009). The CTFs were averaged across permutations and averaged across the four location-specific models. The same weight matrix trained in this fashion on data from 1O trials was used to reconstruct the neural representation of orientation in 1O, 3O, and 1O1C1L trials. More specifically, within each ROI, separate IEMs were trained on 1O data from each time point in the trial and then tested (i.e., reconstructions attempted) at same time point with data from 1O, 3O, and 1O1C1L trials separately. For 3O trials, the location-specific IEM to be used for IEM testing was assigned according to the orientation to be tested for recall. For 1O1C1L trials, testing was conducted with the location-specific IEM congruent with the location occupied by the oriented bar in the sample array.

To quantify the results, the CTF in each ROI for each subject was fit with an exponentiated cosine function of the form:

$$f\left(x\right)\;=\text{α}(e^{\text{k}}[\text{cos}(\text{μ}\;-x)-\text{1}])\;+\text{β}.$$

Here, α and β control the vertical scaling (i.e., signal over baseline) and baseline of the function, respectively, and κ and μ control the concentration (the inverse of dispersion) and center of the function, respectively. No biases in reconstruction centers were expected or observed, so we fixed μ at 0. Fitting was performed by combining a GLM with a grid search procedure. We first defined a range of plausible κ values (from 1 to 30 in 0.1 increments). For each possible value of κ, we generated a response function using the fitting equation after setting α to 1 and β to 0. Next, we generated a design matrix containing the predicted response function and a constant term (i.e., a vector of 1 s) and used ordinary least-squares regression to obtain estimates of α and β (defined by the regression coefficient for the response function and constant term, respectively). We then selected the combination of κ, α, and β that minimized the sum of squared errors between the observed and predicted reconstructions.

In experiment 2, the same IEM procedure was used to estimate neural representations of stimulus orientation and stimulus location, except that orientation models characterized each voxel in terms of the activity in six hypothesized orientation tuning channels (one corresponding to each possible orientation). And six separate IEMs were trained (one for each location) and these location-specific reconstructions were averaged before assessment of the results of IEM training and testing.

#### Implementation of a priori hypothesis tests

Hypothesis 1 and hypothesis 2 of experiment 2 corresponded to analyses from experiment 1 conducted in the occipital ROI and focusing on TR10, one of the TRs at which CTFs in multi-item trials were reliable at the group level (*N *=* *16). To carry out tests of hypotheses 1 and 2 for experiment 2, we first needed to ensure sufficient statistical power to achieve the secondary hypothesis that, for the low swap-error group, the amplitude of the reconstruction of stimulus orientation at TR10, on 3O trials, would be statistically greater than baseline. To do this, we followed our procedure from experiment 1 by assessing the significance of CTF amplitude using a bootstrapping procedure in which, for each ROI, we randomly selected, with replacement, the number CTFs corresponding to the number of subjects in the group, and averaged them. This step was repeated 2500 times, yielding 2500 unique stimulus reconstructions. We then estimated the amplitude of each reconstruction and a *p* value was computed as the proportion of permutations for which amplitude estimates ≤0 were obtained (Ester et al., 2015, 2016). For this and all subsequent bootstrapping analyses, all the *p* values were one-tailed and FDR-corrected across ROIs, TRs, and tasks. Whereas in experiment 1, Cohen’s *d* for the reconstruction of stimulus orientation at TR10 from 3O trials was 0.729, simulations indicated that we could expect effect sizes for reconstruction of stimuli from 3O trials for experiment 2 to double relative to experiment 1, because of the increased number of 3O trials. Therefore, to calculate the number of subjects needed in the low swap-error group to achieve 90% power for this secondary hypothesis, we used an estimate of Cohen’s *d* of [0.729 × 2 = 1.458]. To achieve 90% power to detect this effect with α = 0.025, we needed an *N* of 11. (Note that, because none of the primary hypotheses tested in experiment 2 required significant reconstruction of orientation in the occipital ROI at TR10 in the high swap-error group, we did not carry out power analyses for this effect.)

Additional secondary hypotheses were whether, at any TR of the task, orientation reconstructions differed between the low swap-error and high swap-error groups. These were assessed by first carrying out the bootstrapping procedure separately for each group, then comparing the CTF estimates from each permutation with each group by subtracting the amplitude from the high swap-error group from the corresponding value from the low swap-error group. A one-tailed was conducted with *p* referring to the proportion of the 2500 subtractions with a value ≤0. Finally, we planned to assess the test-retest reliability of *p*N as estimated from the behavioral screening session versus *p*N as estimated from the fMRI session, by Pearson correlation.

Hypothesis 1 of experiment 2 formalized the intuition that one would expect that individual differences in *p*N, a behavioral measure, would be reflected in neural evidence for inappropriate activation of the identity of non-probed items. To assess this prediction, as we did in experiment 1, we operationalized “orientation recall specificity” as the difference (on 3O trials) between probe-epoch CTFs of the orientation of the probed versus of a non-probed item, then compared this measure between swap-error groups. To compute orientation recall specificity, for each trial one of the two non-probed sample items was selected at random and the amplitude of the reconstruction of the orientation of that non-probed item was subtracted from the amplitude of the reconstruction of the orientation of the probed item. Significance of all comparisons was assessed with bootstrapping. Using the results from experiment 1 as a starting point, and taking into account that fact that the modified design of experiment 2 increased effect sizes for reconstruction of stimuli from 3O trials, we determined that we required an *N* of 10 subjects to achieve 90% power to detect an orientation recall-specificity effect with α = 0.025 (two-tailed *t* test).

Hypothesis 2 of experiment 2 was analogous to hypothesis 1, with the difference being that it operationalized the specificity of recall of the location of the probed item. Thus, we operationalized “location recall specificity” as the difference between probe-epoch CTFs of the location of the probed versus of a non-probed item, then compared this measure between swap-error groups. To compute location recall specificity, for each trial one of the two non-probed sample items was selected at random and the amplitude of the reconstruction of the location of this non-probed item was subtracted from the amplitude of the reconstruction of the location of the probed item. Significance of all comparisons were assessed with bootstrapping. Because experiment 1 did not use IEM to assess the neural representations of stimulus location, we used a different dataset from our lab to estimate power, and these data suggested that data from only three subjects were needed to achieve 90% power to detect a reliable location recall-specificity effect with α = 0.025 (two-tailed *t* test).

Results from experiment 1 suggested that delay-period representation of sample locations in parietal cortex was stronger in the low swap-error group. Specifically, MVPA decoding of stimulus location was superior for the low swap-error group at TR6 and TR7. Follow-up correlational analyses indicated that individual differences in MVPA of sample location information in parietal delay-period activity correlated with *p*N and with the location recall specificity effect. Hypothesis 3 was therefore intended to assess the prediction that individual differences in the strength of delay-period representation of the sample location in parietal cortex, as operationalized by the amplitude of the CTF of the location of the to-be-probed item at TR6 and TR7, would predict individual differences in *p*N. We planned to carry this out with Spearman correlation of rank ordering of these two variables, because subjects in the low swap-error group would necessarily have *p*N values close to 0. The coefficient of determination (*R*^2^) for this effect in experiment 1, with an *N* of 16, was 0.282. Because hypothesis 4 would also use the same measure of delay-period representation of the sample location in parietal cortex, we set α = 0.025 and calculated that, to achieve 90% power to find this effect, we would need to enroll an *N* of 29.

Hypothesis 4 assessed the prediction that individual differences in the strength of delay-period representation of sample locations in parietal cortex, as operationalized by the amplitude of the CTF of the location of the to-be-probed item, would predict individual differences in location recall specificity. We planned to carry this out with a Pearson correlation, because we expected, based on experiment 1, that both variables would be normally distributed. (However, if either distribution turned out to be skewed (as estimated via Shapiro–Wilk analysis), we would instead use Spearman correlation). Based on the fact that the *R*^2^ for this effect in experiment 1, with an *N* of 16, was 0.303, we estimated that we would need *N *=* *25 to achieve 90% power to detect this effect at α = 0.025.

## Results

### Experiment 1

The only results from experiment 1 that are reported are those that were not used to generate the preregistered hypotheses, which were tested in experiment 2.

#### Behavioral session

Here, we report results from only the 16 subjects who also participated in the fMRI experiment. An average of 2.286 trials (SD = 2.920); 4.857 trials (SD = 2.824), and 1.857 trials (SD = 2.824) per subject were excluded in the 1O, 3O, and 1O1C1L condition, respectively. Descriptive statistics suggested that task difficulty increased from 1O to 1O1C1L to 3O, as reflected in the mean response error (*F*_(2,30)_ = 41.830, *p* < 0.0001; paired *t* test, *t*s* *>* *3.960, *p*s <* *0.002; Table 1). Results from mixture modeling mirrored this pattern, with pT highest for 1O, followed by 1O1C1L and 3O (*F*_(2,30)_ = 16.791, *p* < 0.0001), paired *t* test *t*s* *>* *2.644, *p*s <* *0.018; Table 1). Recall precision (κ) also differed across trials types (*F*_(2,30)_ = 16.458, *p *< 0.001), being significantly different between 1O and 3O (*t*_(15)_ = 5.253, *p* < 0.0001) and between 3O and 1O1C1L (*t*_(15)_ = 4.325, *p *<* *0.001) trials, although not differing between 1O and 1O1C1L trials (*t*_(15)_ = 1.387, *p *=* *0.186). Finally, although the group mean *p*N (swap errors) on 3O trials was 0.120 (SD = 0.116), six subjects had a *p*N at or near 0 (all *p*Ns < 0.006), indicating that these subjects made effectively no swap errors, whereas the remaining 10 subjects all had a *p*N of 0.127 or higher, corresponding to an average of nearly 20% swap errors on 3O trials for these ten subjects.

**Table 1:** Behavioral results from experiment 1 (*n* = 16 for both sessions)

	Descriptive data		Three-factor mixture model (parameter estimates)			
Trial type	RT(s)	Response error (degree)	*p*T	*p*N	*p*U	κ(rad^−1^)
Behavior-only session						
1O	2.825 (0.247)	7.395 (2.360)	0.987 (0.019)	N/A	0.013 (0.019)	12.899 (7.706)
3O	2.941 (0.231)	16.214 (5.889)	0.828 (0.120)	0.120 (0.116)	0.052 (0.064)	4.166 (2.230)
1O1C1L	2.687 (0.336)	10.764 (5.076)	0.895 (0.114)	N/A	0.105 (0.114)	10.718 (7.004)
fMRI session						
1O	2.912 (0.149)	7.765 (4.219)	0.946 (0.001)	N/A	0.054 (0.001)	13.748 (6.881)
3O	3.029 (0.223)	16.510 (7.308) --	--	--	--	
1O1C1L	3.009 (0.248)	12.284 (4.028) --	--	--	--	

#### fMRI sessions

##### Behavior

An average of 4.62 (SD = 2.07) 1O trials was excluded, no trials for any subject for either of the other two trial types were excluded. Performance on 1O trials was highly correlated with pre-scan testing across the 16 subjects who participated in both (*r*s* *>* *0.841, *p*s* *<* *0.001; Table 1).

##### fMRI

For the occipital ROI, trial-averaged signal on all three trial types showed the expected sample-related increase, return to baseline by the end of the delay period, and probe/recall-related increase. BOLD signal intensity did not differ between 3O and 1O1C1L trials during TRs corresponding to the encoding and delay epochs (from TR2–TR8, *t*s* *<* *1.604, *p*s* *>* *0.130), but was higher on 3O than 1O1C1L trials during the peak response to probe/recall (TR9–TR11, *t*s* *>* *2.524, *p*s* *<* *0.048). BOLD signal from all three trial types did not differ from each other or from baseline at TR7 corresponding to late delay (*t*s* *<* *0.806, *p*s* *>* *0.433; Fig. 2*A*). The ANCOVA relating delay-period BOLD signal intensity to behavioral precision revealed no significant within-subject correlations between either 1O and 3O trials or 1O and 1O1C1L trials (Fig. 2*B*).

For the parietal and frontal ROIs, in both regions, trial-averaged signal on all three trial types remained elevated across the duration of the trial, with sample-related and delay-related activity greater for 3O trials than for the other two trial types (from TR3 to TR8, *t*s* *>* *3.041, *p*s* *<* *0.019), and sample-related activity for 1O1C1L trials greater than for 1O trials during the encoding epoch (TR3–TR4, *t*s* *>* *2.514, *p*s* *<* *0.048). Beginning with TR6 in the delay period, however, BOLD signal no longer differed between 1O1C1L and 1O trials (*t*s* *<* *1.848, *p*s* *>* *0.138). That is, delay-period activity in parietal and frontal cortex was not sensitive to memory load (one item vs three items) per se, but was sensitive to stimulus category homogeneity (operationalized as 1O1C1L vs 3O; Fig. 2*A*). ANCOVAs relating delay-period BOLD signal intensity to behavioral precision revealed significant within-subject correlations between 1O and 3O trials in both regions (*r*s* *>* *0.539; *p*s* *<* *0.05), and a nonsignificant trend in this direction between 1O1C1L and 3O trials in parietal cortex (*r *=* *0.458; *p* = 0.06. Fig. 2*B*).

### Experiment 2

#### Behavioral

##### Behavioral session

A total of 39 of 72 subjects who met a behavioral screening criterion of performing with a “low” swap error rate (<0.05) or “high” swap error rate (>0.12) were invited to participate two subsequent fMRI sessions. From these, 29 subjects completed the two fMRI scanning sessions [low swap-error group, mean(SD): *p*T* *=* *0.923(0.085), *p*N* *=* *0.015(0.015), *p*U* *=* *0.062(0.080), κ = 3.844(2.402); high swap-error group, *p*T* *=* *0.674(0.133), *p*N* *=* *0.254(0.085), *p*U* *=* *0.071(0.075), κ = 3.294(1.815)].

##### fMRI session

Two subjects were excluded from the fMRI analyses – one because responses were not registered on >40% trials, and one because mixture modeling indicated that *p*U was >0.75 (outside three SD from the mean value), and so the data from 27 are reported here. The behavioral performance of many of these 27 subjects, specifically their *p*N, differed considerably during the scanning sessions in comparison to the behavioral screening session, with eight subjects initially classified as “low swap error” having *p*N values > 0.05 and one subject initially classified as “high swap error” having a *p*N value < 0.12. A consequence of this that the same sample of subjects who had been selected to form a bimodal distribution of “extreme” values of *p*N yielded a continuous distribution of values from the fMRI session, with 15 subjects with *p*N values < 0.05, six with *p*N values > 0.05 and < 0.12, and eight with *p*N values > 0.12 (Fig. 2). As a consequence, with too few high swap-error subjects, we were unable to test our hypotheses with the preregistered analyses. We reasoned, however, that the original intent of the preregistered hypothesis tests would be met if we deviated from the preregistered criteria for classifying subjects and, instead, conducted the planned analyses on two groups determined by a median split of *p*N values. From the perspective of scientific publishing, this deviation from the preregistered plan would qualify these analyses as “exploratory” were this a formal Registered Report. From the perspective of our experimental design, it might be expected a priori to decrease the power of the design, because several subjects in the high swap-error group had with *p*N values lower than the lower bound of *p*N* *> 0.12. that had been used to calculate power.

#### Analyses of data from fMRI session grouped by median split

**Figure 3.**
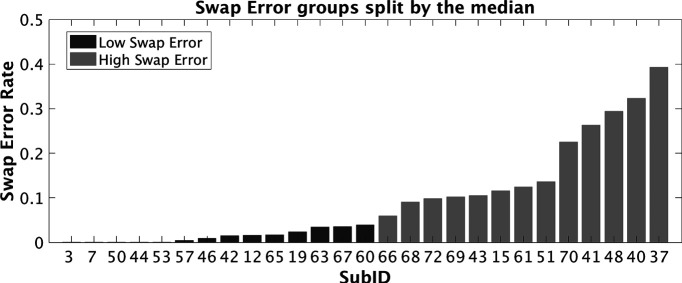
Swap error rates for each subject in the fMRI session of experiment 2. Low swap-error subjects (dark gray) and high swap-error subjects (light gray) were defined via median split.

The median split assigned 14 subjects, with *p*Ns ranging from 0 to 0.039, to the low swap-error group (mean(SD): *p*T* *=* *0.896(0.127), *p*N* *=* *0.012(0.013), *p*U* *=* *0.091(0.125), and κ = 3.092 (1.498)], and 13 subjects, with *p*Ns ranging from 0.059 to 0.393, to the high swap-error group [mean(SD): *p*T* *=* *0.700(0.170), *p*N* *=* *0.169(0.109), *p*U* *=* *0.131(0.177), and κ = 3.652(1.881); Fig. 3). Independent *t tests* between the groups revealed no significant difference in *p*U or κ (*t*s < 0.851, *p*s* *>* *0.403). Test-retest stability between the behavioral-screening and fMRI sessions was assessed with correlation analyses. Pearson correlations revealed significant test-retest correlations for *p*T and for κ (*R*^2^ > 0.477, *p*s <* *0.001). Because *p*N from the behavioral screening session was selected to be dichotomous, a Spearman correlation was conducted on this measure, and it failed to provide evidence for a correlation of performance across the two sessions (*R*^2^ = 0.054, *p *=* *0.247).

#### Hypothesis tests

##### Hypothesis 1

**Figure 4.**
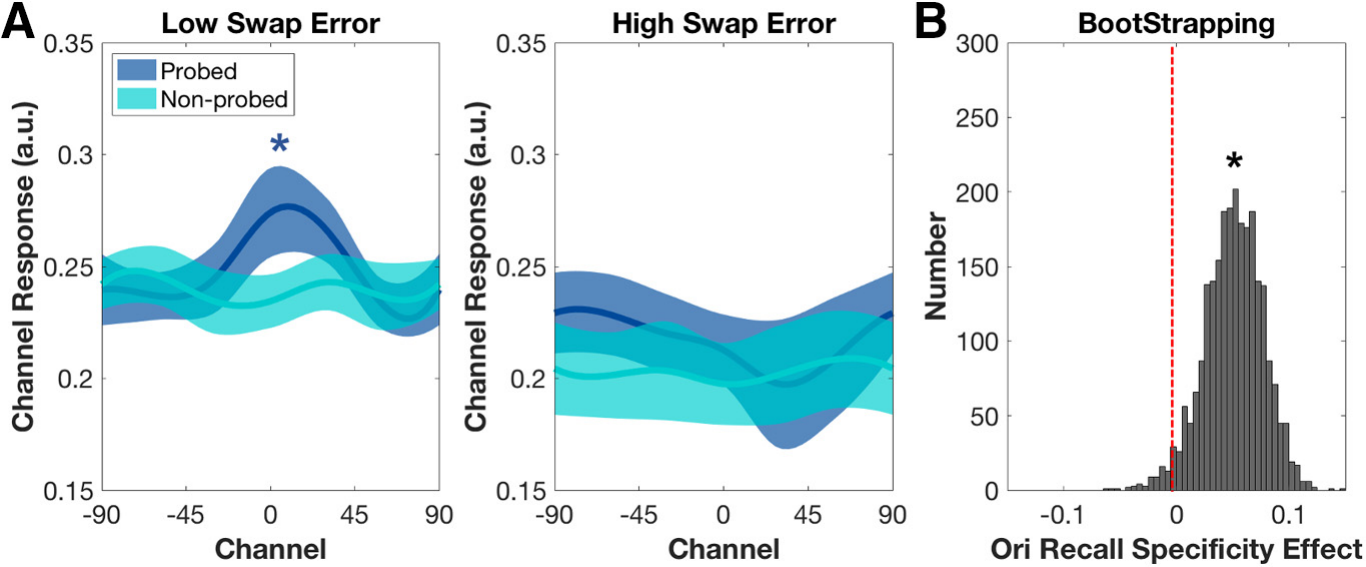
Orientation recall specificity effect in the occipital ROI, at TR10 on 3O trials (experiment 2). ***A***, Neural reconstructions for probed and non-probed orientations in the two groups. Asterisks indicate significant reconstructions at *p *<* *0.05. ***B***, Distributions of bootstrapping estimates of orientation recall-specificity effects. The recall specificity effects were identified by the recall specificity difference between low and high swap error group. Asterisk indicates significant difference between the two distributions (*p *<* *0.05).

In the occipital ROI, at TR10, for the low swap-error group, IEM reconstruction of stimulus orientation on 3O trials was significantly larger than baseline (*p *=* *0.047; Fig. 4*A*), thereby meeting the prerequisite for being able to test hypothesis 1. (Neither the comparable result for the high swap-error group, nor the reconstructions of the orientation of a non-probed item for either group, approached significance; *p*s* *>* *0.671; Fig. 4*A*). Comparison of orientation recall specificity between the two groups indicated that this measure was higher for the low swap-error group than for the high swap-error (*p *=* *0.049; Fig. 4*B*), and thus supported hypothesis 1.

##### Hypothesis 2

**Figure 5.**
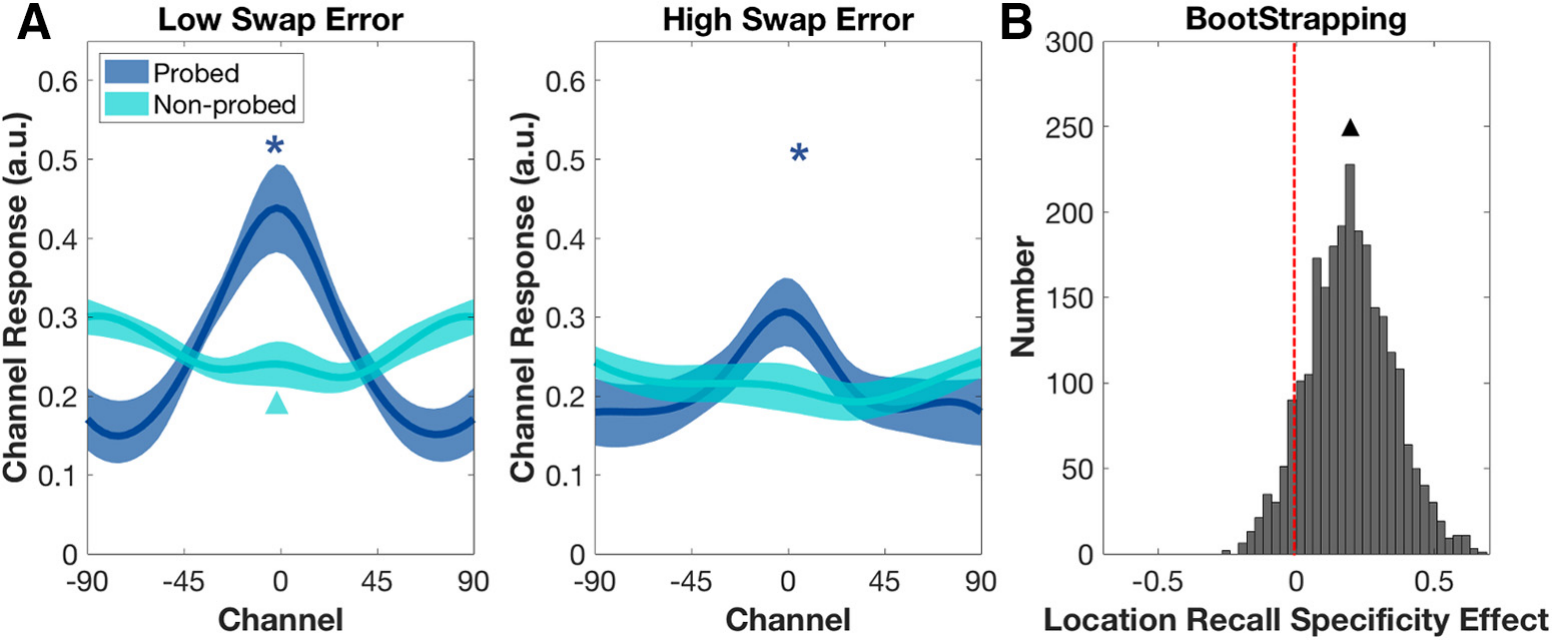
Location recall specificity effect in the occipital ROI, at TR10 on 3O trials (experiment 2). ***A***, Neural reconstructions for probed and non-probed locations in the two groups. Asterisks indicate significant reconstructions (*p *<* *0.05), and triangle indicates trend-level evidence for a significantly negative-going reconstruction (0.05 < *p* < 0.1). ***B***, Distributions of bootstrapping estimates of location recall-specificity effects in the two groups. Triangle indicates trend-level evidence for a difference between the two distributions (0.05 < *p* < 0.1).

In the occipital ROI, at TR10, IEM reconstruction of the probed location on 3O trials was significantly larger than baseline for both the low swap-error group (*p *<* *0.001) and the high swap-error group (*p *=* *0.021; Fig. 5*A*). Comparable reconstructions for non-probed locations were nonsignificant for both groups (low swap error, *p *=* *0.059 (trending toward negative); high swap error *p *=* *0.770; Fig. 5*A*). Comparison of location recall specificity between the two groups indicated that although this measure was numerically higher for the low swap-error group than for the high swap-error group (Fig. 5*B*), this difference approached, but did not achieve, the threshold for significance (*p *=* *0.067).

##### Hypothesis 3

IEM did not yield reliable reconstruction of the to-be-probed location in the parietal ROI during TR6 and TR7 of the delay period for either group (*p*s >* *0.213), and so a direct test of hypothesis 3, as it was preregistered, could not be conducted. (Note that the exclusion of two subjects was not factor here, because the planned *N* of 29 was needed for the planned correlation, not for the reliable reconstruction of the to-be-probed location, which would have been expected to require considerably fewer subjects). When we nevertheless conducted the planned Spearman correlation with data from TR6 and TR7, it was not significant (*p* > 0.23). However, because the reconstruction of stimulus location was significant for the low swap-error group at adjacent TR8 (*p* = 0.041), we applied the procedures for testing hypothesis 3 to this adjacent time point, as an exploratory analysis. Location representation-specificity was reliable at TR8 for the low swap-error group (*p *=* *0.041) but not for the high swap-error group (*p* = 0.566). Across all subjects, Spearman correlation indicated that higher values of location representation-specificity in IPS were associated with lower values of *p*N (*R*^2^ = 0.269, *p *=* *0.01).

##### Hypothesis 4

For the same reason as hypothesis 3, a direct test of hypothesis 4, as it was preregistered, could not be conducted. Thus, analogous to hypothesis 3, we also applied the procedures for testing hypothesis 4 to TR8, as an exploratory analysis. Across all subjects, Spearman correlation indicated that higher values of location representation-specificity in IPS were associated with higher values of location recall specificity in occipital cortex, at TR10 (*R*^2^ = 0.357, *p *=* *0.001).

#### Secondary hypotheses

##### BOLD signal intensity

In all three ROIs, for 1O and 3O trials, the time course of fMRI activity was comparable to what was observed in experiment 1: In the occipital ROI, at TR7, signal in the occipital ROI did not differ between 3O and 1O trials (*t*_(26)_ = 1.617, *p *=* *0.118); whereas it was significantly higher for 3O than for 1O trials in the parietal and frontal ROIs (*t*s > 3.572, *p*s <* *0.001). Furthermore, the ANCOVAs relating load-related changes in signal at TR7 to load-related changes in behavioral recall precision was not significant in the occipital ROI, but the analogous ANCOVAS were significant in both the parietal (*R*^2^ = 0.48) and frontal (*R*^2^ = 0.4; *p*s <* *0.001) ROIs.

##### The time course of IEM reconstructions of orientation during 3O trials

**Figure 6.**
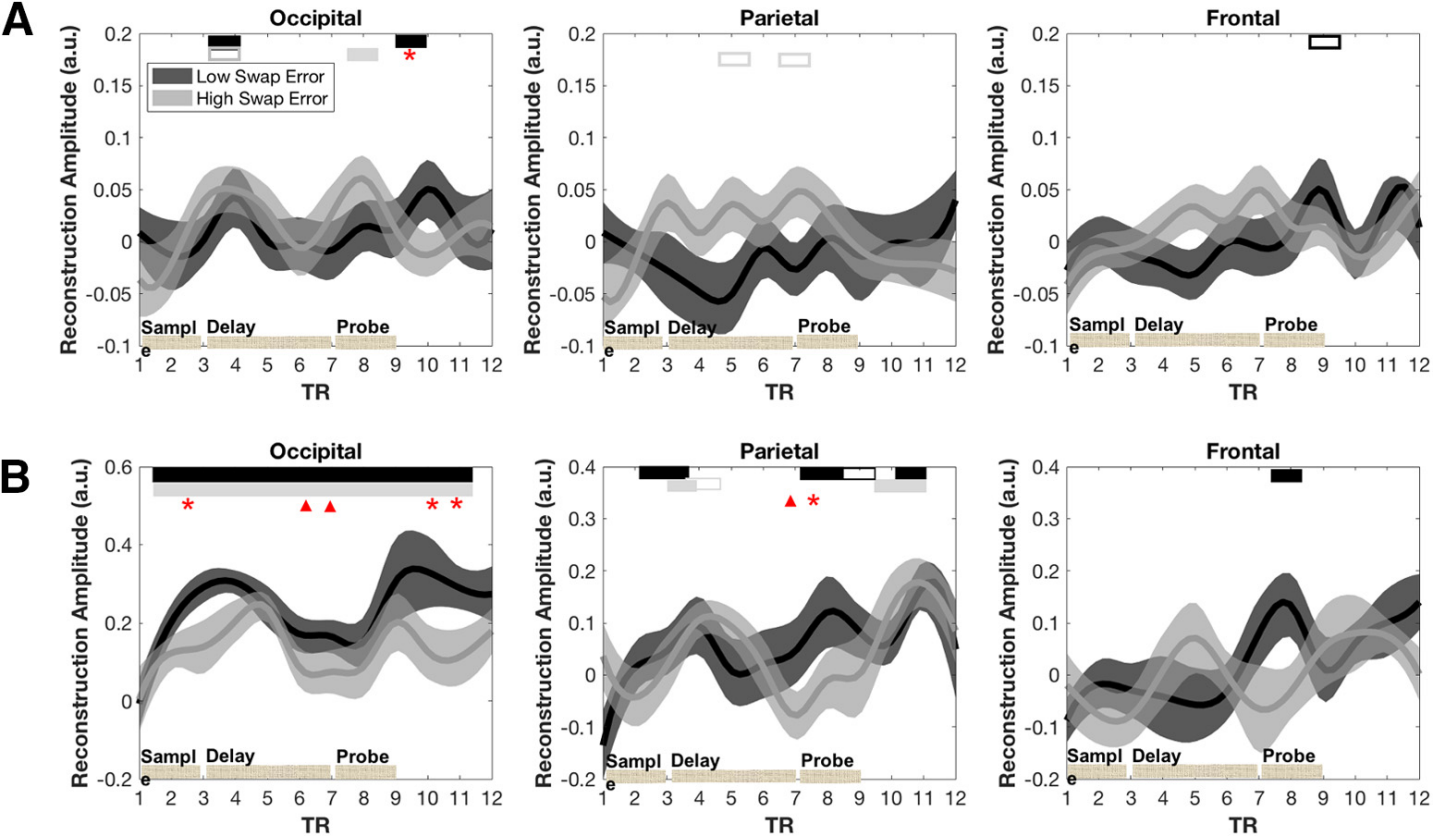
Time course of IEM reconstructions of unshifted data from 3O trials. ***A***, Reconstructions of the orientation of the probed stimulus broken out by swap-error group, in the three ROIs. ***B***, Reconstruction of the location of the probed stimulus broken out by swap-error group, in the three ROIs. Bars along the top indicate values statistically different from baseline, with color corresponding to swap-error group; * indicates statistical difference between swap-error group at that TR (not FDR-corrected). Bars along the bottom indicate the timing of trial epochs.

In the occipital ROI, the reconstruction of the to-be-probed orientation was successful during the sample-presentation and recall epochs (low swap-error group: TR4 and TR10; high swap-error group: TR4 and TR8; *p* values for these IEM reconstructions not FDR-corrected), but not during the delay period. Orientation could not be reconstructed in the parietal and frontal ROIs (Fig. 6*A*). (Note that it has previously been reported that, on 1O trials, when collapsing across all subjects, orientation can be reconstructed during the entirety of the trial in each of these three regions; Cai et al., 2019.)

Turning to the reconstruction of the location of the probed item, in the occipital ROI location could be reconstructed across the entirety of the trial in both groups, with reconstruction amplitude significantly greater for the low swap-error group during portions of sample presentation (TR3) and recall (TR10 and TR11) epochs, and trending in this direction during the delay period (TR6 and TR7; Fig. 6*B*). In the parietal ROI, location could be reconstructed during the sample presentation (TR3 and TR4) and during recall (TR8–TR11) in both groups, with a trend toward greater amplitude in the low swap-error group at TR7 and TR8 (Fig. 6*B*). In the frontal ROI location reconstruction was only successful at one time point during recall (TR8) in the low swap-error group (Fig. 6*B*).

## Discussion

Success on VWM tasks that are nominally nonspatial can often nonetheless require memory for the location at which each item was presented, and thus require context binding (compare Olson and Marshuetz, 2005; Jiang et al., 2009). The results presented here are consistent with the proposition that delay-period activity of the IPS may correspond more closely to the operation of context binding than to the representation of stimulus identity per se. Experiment 1 varied the demands on context binding by using two types of three-item trials, 3O trials that could only be solved if the subject remembered which item had been presented at the location of the recall dial, and 1O1C1L trials in which memory for item-location associations was not needed, because the nonspatial features of the recall dial uniquely indicated which item was to be recalled. The delay-period BOLD response in IPS (and in frontal cortex) was sensitive to demands on context binding (3O vs 1O1C1L), but not to the number of items that needed to be remembered (1O vs 1O1C1L; Fig. 2). This finding replicates and extends a previous finding (Gosseries et al., 2018), and has at least two implications for our understanding of VWM. First, it suggests that the interpretation of results from many previous reports of load-sensitive activity in IPS (Todd and Marois, 2004, 2005; Xu and Chun, 2006) may need to be revisited, because of the possibility that such studies have often confounded the factors of load and of context binding. Second, it provides an explicit, mechanistic account of the “benefit of memory set heterogeneity” (Olson and Jiang, 2002; Delvenne and Bruyer, 2004), and may provide support for models that explicitly incorporate context binding (Swan and Wyble, 2014; Oberauer and Lin, 2017; Bouchacourt and Buschman, 2019) as well as a challenge for models that do not (including slot and resource models; compare Oberauer et al., 2016).

The results from experiment 2 broadly replicated the intriguing preliminary findings from experiment 1 that individual differences in the rate of swap errors were associated with neural measures of the strength of the representation of the (nonspatial) orientation and the location of stimuli. Low swap-error subjects showed higher orientation recall specificity in occipital cortex (hypothesis 1), and a trend in the same direction for location recall specificity (hypothesis 2). These results provide a demonstration, at the neural level, for a phenomenon predicted by a theoretical and computational model of VWM: stronger context binding should result in a lower level of competition between the probed item and nonprobed items at the time of retrieval (Oberauer and Lin, 2017).

For IPS, exploratory results from experiment 2 showed a pattern comparable to what was seen experiment 1, but at a later time point: the strength of delay-period representation of stimulus location (i.e., of context) in IPS predicted lower swap-error rates (hypothesis 3), and higher location recall specificity in occipital cortex (hypothesis 4). These are consistent with the proposal from Gosseries et al. (2018) that delay-period activity in IPS may be particularly important for the maintenance of the bindings of context to content. We note that whereas the present results are consistent with the proposed functions of a spatial priority map (Bisley and Goldberg, 2010; Jerde et al., 2012; Bisley and Mirpour, 2019), the task-critical context in Gosseries et al. (2018) was ordinal position. Thus, an important question for future research is determining whether the processing of context in IPS is conducted in a domain-specific or a domain-general manner.

Interpretation of some of the results from experiment 2 is complicated by a pattern in the results of this multisession experiment that was unanticipated: subject’s swap-error rates (*p*N) were less stable between the behavioral screening session and the fMRI sessions than were other indices of performance, including precision (κ) and the probability of a target response (*p*T). As a result, several subjects selected for the low swap-error group based on performance at behavioral screening performed the task with a higher rate of swap errors during the fMRI sessions. The resultant decrease in sensitivity relative to what had been expected for the preregistered extreme-groups design might explain why the between-group difference in location recall specificity did not achieve statistical significance. There are at least two possible explanations for the greater volatility of estimates of *p*N than those of κ and of *p*T. The less interesting possibility is a technical one: model estimates of *p*N may simply be less robust than those of other parameters, particularly at the relatively small number of trials that features in the fMRI sessions from experiment 2. (Note, however, that we did try two other models that we only became aware of after preregistering experiment 2 (Bays, 2016; Oberauer et al., 2017), but these did not produce appreciably different results). The potentially more interesting possibility is that the hypothesized mechanism that is indexed by *p*N, context binding, may be more sensitive to challenging testing conditions (e.g., the MRI scanner session vs the behavioral lab) than are the mechanisms underlying κ and *p*T.

## Synthesis

Reviewing Editor: Morgan Barense, University of Toronto Decisions are customarily a result of the Reviewing Editor and the peer reviewers coming together and discussing their recommendations until a consensus is reached. When revisions are invited, a fact-based synthesis statement explaining their decision and outlining what is needed to prepare a revision will be listed below. The following reviewer(s) agreed to reveal their identity: Klaus Oberauer.

I was able to secure one reviewer from your original Journal of Neuroscience submission, and I also reviewed the manuscript itself. The reviewer and I agree that this is an excellent paper. In the words of the reviewer: “This is one of the best revisions I have ever reviewed. The manuscript was already strong in the first version. The preregistered replication included in the present revision allays all my concerns about the limited credibility of some results from Experiment 1.”

We see the major advances as threefold:

(1) This is one of the first studies to investigate the neural signatures of item-context bindings in working memory. Recent theoretical work (cited in the manuscript) has emphasized the important role of item-context bindings for working memory, but the cognitive neuroscience of working memory (WM) has so far focused on the (easier to measure) neural correlates of item representations. Therefore, the present work is a significant step forward.

(2) The authors use inverted encoding models to measure the precision of representations of both memory contents (orientations) and contexts (locations), and link these measures to intra- and inter-individual differences in behavior, thereby making a first step towards validating the neural measures.

(3) The finding that both behavioral accuracy and the BOLD signal strength track not only the number of items in WM but also their homogeneity - and thereby, the degree of interference between them - is a highly diagnostic finding for adjudicating between competing theories of WM: Neither slot models nor continuous resource models predict such an effect of item heterogeneity (Oberauer, Farrell, Jarrold, & Lewandowsky, 2016). The reviewer suggested that this aspect of the results could be emphasized more in the manuscript.

We have only a few minor comments that we hope will help the authors to improve the manuscript in a revision:

- Line 59: The IPS signal is described as being larger in 3M than 1M but also comparable - is this a typo?

- Line 96: “increase [...] power to detect effects by reducing the variance between participant groups” - should this not be “increase”?

- Line 100 onwards: The hypotheses are virtually impossible to understand as stated here (for instance, readers don’t know yet what “orientation recall specificity effect” means, and why TR 10 is relevant for this hypothesis). Perhaps this could come later in the manuscript, at a point where these concepts have been introduced, or else, the hypotheses could be explained here in a way that can be understood.

- The paragraph starting in line 192 is redundant.

- Why was the IEM trained separately for each location? In my understanding, the IEM is essentially a regression model, and estimates of regression-model parameters benefit from variability in the criterion, which would be much higher if all locations were included in one model.

- The reviewer still expressed confusion about the ANCOVAs: What variable was correlated with what? For instance, line 438 ff describes an ANCOVA looking at the relation between BOLD signal and behavioral precision across pairs of conditions. Is the BOLD signal correlated with behavioral precision separately in each condition, or is the BOLD signal from one condition correlated with the BOLD signal from the other condition, ...?

- Line 536: Why is it important to ensure that the reconstruction amplitude is significant for the low-swap group? It would be helpful to explain this.

- Line 686: The 3 mixture weights add up to >1, which is probably because the PT is estimated from the 1O trials, whereas PN is from the 3O trials. Still, this is confusing - report the parameter estimates for each condition separately.

- The median split by pN from the scanning session is a reasonable decision in light of the lack of correlation of pN across sessions. An alternative approach would be to use the first-session pN variables to define the groups. As this had to become an exploratory analysis anyway, it would be good to run it both ways to gauge the robustness of the results across two equally reasonable approaches.

- Line 754: Selecting TR by whether the location reconstruction was significant for low-swap subjects biases the correlation in the direction of the hypothesis because it potentially selects a TR in which, simply due to noise, the location specificity of the low-swap subjects (but not the high-swap subjects) was elevated. Therefore, it would be reassuring to see the correlation also reported for the originally planned TR7.

- In Figure 7, was the neural signal shifted back in time by the average BOLD delay?

- The second sentence of the abstract contains three “versus” statements, making it a bit challenging to unpack what is being compared.

## Author Response

I was able to secure one reviewer from your original Journal of Neuroscience submission, and I also reviewed the manuscript and your rebuttal letter. The reviewer and I agree that this is an excellent paper. In the words of the reviewer: “This is one of the best revisions I have ever reviewed. The manuscript was already strong in the first version. The preregistered replication included in the present revision allays all my concerns about the limited credibility of some results from Experiment 1.”

> yay

We see the major advances as threefold:

(1) This is one of the first studies to investigate the neural signatures of item-context bindings in working memory. Recent theoretical work (cited in the manuscript) has emphasized the important role of item-context bindings for working memory, but the cognitive neuroscience of working memory (WM) has so far focused on the (easier to measure) neural correlates of item representations. Therefore, the present work is a significant step forward.

(2) The authors use inverted encoding models to measure the precision of representations of both memory contents (orientations) and contexts (locations), and link these measures to intra- and inter-individual differences in behavior, thereby making a first step towards validating the neural measures.

(3) The finding that both behavioral accuracy and the BOLD signal strength track not only the number of items in WM but also their homogeneity - and thereby, the degree of interference between them - is a highly diagnostic finding for adjudicating between competing theories of WM: Neither slot models nor continuous resource models predict such an effect of item heterogeneity (Oberauer, Farrell, Jarrold, & Lewandowsky, 2016). The reviewer suggested that this aspect of the results could be emphasized more in the manuscript.

> To do this, we have modified the end of the first paragraph of the discussion, as follows (new text underlined):This finding replicates and extends a previous finding (Gosseries, Yu, et al., 2018), and has at least two implications for our understanding of visual working memory. First, it suggests that the interpretation of results from many previous reports of load-sensitive activity in IPS (e.g., Todd and Marois 2004, Todd and Marois 2005, Xu and Chun 2006) may need to be revisited, due to the possibility that such studies have often confounded the factors of load and of context binding. Second, it provides an explicit, mechanistic account of the “benefit of memory set heterogeneity” (e.g., Olson and Jiang 2002, Delvenne and Bruyer 2004), and may provide support for models that explicitly incorporate context binding (e.g., Swan and Wyble 2014, Oberauer and Lin 2017, Bouchacourt and Buschman 2019) as well as a challenge for models that do not (including slot and resource models, c.f., Oberauer et al. 2016).

We have only a few minor comments that we hope will help the authors to improve the manuscript in a revision:

- Line 59: The IPS signal is described as being larger in 3M than 1M but also comparable - is this a typo?

The sentence in question reads “Although delay-period activity of the IPS was elevated during 3M relative to 1M trials, it was comparable for 1M2C and 1M trials, indicating sensitivity to a factor other than memory load per se.” There are no typos, this reads as intended.

- Line 96: “increase [...] power to detect effects by reducing the variance between participant groups” - should this not be “increase”?

Here the phrase in question is a direct quote from Yarkoni and Braver (2010). For context, the full phrase is: “a priori extreme groups design can be an effective way to “increase [...] power to detect effects by reducing the variance between participant groups relative to the variance within groups, thereby inflating effect sizes and making them easier to detect (Yarkoni and Braver 2010).”” We have double checked and confirm that the manuscript is verbatim with how it appears in the original.

- Line 100 onwards: The hypotheses are virtually impossible to understand as stated here (for instance, readers don’t know yet what “orientation recall specificity effect” means, and why TR 10 is relevant for this hypothesis). Perhaps this could come later in the manuscript, at a point where these concepts have been introduced, or else, the hypotheses could be explained here in a way that can be understood.

We fully understand the sentiment, but call attention to two factors that argue against changing anything. First, the convention for Registered Reports is to state the preregistered hypotheses in the Intro. or first-thing in the Methods, presumably to emphasize their centrality to the mechanism. Second, the journal’s guidelines explicitly forbid changing the wording of preregistered hypotheses once they are approved and, indeed, specify that making any changes to them is grounds for rejection. Without wanting to come across as being unresponsive, we don’t believe there’s a way to restate these hypotheses “in a way that can be understood” short of simply moving them to the end of the Methods section. Many of the details of the Methods are necessary to be able to make sense of the final subsection of the Methods called Implementation of a priori hypothesis tests, and this section itself is 93 lines long. We tried to allay this concern in our initial submission with the sentence that appears after Hypothesis 4. In order to make this message preemptive, we’re proposing to also include a footnote at the end of the first hypothesis: “Note that for Registered Reports the convention is to state the preregistered hypotheses in the Introduction or the very beginning of the Methods section. As a consequence, explanation of the rationale behind specific predictions, and of the names of some of the measures in which the hypotheses are framed, appear in a subsection at the end of the Methods section, titled Implementation of a priori hypothesis tests.” Update: After submitting this revision with this footnote included, the manuscript was flagged by the Initial Quality Check because the journal formatting rules don’t permit footnotes. And so we've decided to move this text into a parenthetic ‘disclaimer’ that appears directly before the statement of the hypotheses. This makes for an awkward discontinuity in the narrative flow, but we think it preferable to moving the statement of the hypotheses to the very end of the Methods section, which would, in our view, be incongruent with the fact that these hypotheses were preregistered, prior to carrying out Experiment 2.

- The paragraph starting in line 192 is redundant.

deleted

- Why was the IEM trained separately for each location? In my understanding, the IEM is essentially a regression model, and estimates of regression-model parameters benefit from variability in the criterion, which would be much higher if all locations were included in one model.

Previous analyses on a subset of these data (only the 1O trials from Exp. 1), published as Cai et al. (2019), indicated that models (of orientation) trained separately were more robust then “all locations” models, and furthermore only the reconstructions of orientation with location-dependent models predicted (behavioral) recall precision.

- The reviewer still expressed confusion about the ANCOVAs: What variable was correlated with what? For instance, line 438 ff describes an ANCOVA looking at the relation between BOLD signal and behavioral precision across pairs of conditions. Is the BOLD signal correlated with behavioral precision separately in each condition, or is the BOLD signal from one condition correlated with the BOLD signal from the other condition, ...?

In Fig. 2B there are four plots. In the left-most, BOLD signal from IPS is negatively correlated with behavioral precision across two levels of stimulus type: at load 3, BOLD is high and precision is low, whereas at load 1, BOLD is low and precision is high. The ANCOVA removes the variance attributable to individual differences, so in the plots, the slope of the regression line is how those to variables relate within this sample, and the offset between colored lines, although factored out in the analysis, shows where individual subjects sit in this BOLDxBehavior space. (Note that the fact that each subject contributes two values prevents us from using more conventional regression.) In the second plot, BOLD signal from the frontal ROI is negatively correlated with behavioral precision across these two levels of stimulus type. In the third plot, IPS is negatively correlated with behavioral precision across two different levels of stimulus type: 3O vs. 1O1C1L. In the fourth, BOLD signal from the frontal ROI is negatively correlated with behavioral precision across 3O vs. 1O1C1L.

Because it seems that this level of “walk-through” would be helpful, we have added this sentence to the figure legend for 2.B: “Thus, for example, the left-most plot illustrates that on 3O trials, delay-period BOLD signal in IPS is high and precision is low, whereas on 1O trials, delay-period BOLD signal in IPS is low and precision is high. The slope of the lines indicates the tendency at the group level, and the offsets between lines indicate where different individuals sit within this space.”

The axes are correctly labeled (BOLD vs. precision on each) and this analysis and this graphical depiction of it has been published by us twice previously (Emrich et al, 2013; Gosseries et al., 2018; both cited). We did note, while reinspecting everything to put together this response, that the figure legend for Fig. 2B contained a mistake in referring to one class of symbol in the plots, and we have fixed that.

- Line 536: Why is it important to ensure that the reconstruction amplitude is significant for the low-swap group? It would be helpful to explain this.

This is a criterion that was (correctly in our view) articulated by one of the reviewers of the original J. Neurosci. submission. The reasoning is that if we want to use the orientation recall specificity effect as an index of the robustness of orientation recall operations to “intrusion” from nontargets, we need to first establish that orientation recall is happening. Without evidence for the presence of the first-order phenomenon of stimulus reconstruction, we’d have no basis for asserting that the more involved analyses (of the same data) that go into computing the orientation recall specificity effect actually reflect the efficiency of recall.

- Line 686: The 3 mixture weights add up to >1, which is probably because the PT is estimated from the 1O trials, whereas PN is from the 3O trials. Still, this is confusing - report the parameter estimates for each condition separately.

This section is reporting results from the Behavioral Screening Session, and so only 3O trials were administered. But the reviewer did, indeed, catch a typo, in that the correct value of PT was actually .923 (not .993) and so now the three mixture weight do add up to 1 (slightly over due to rounding).

- The median split by pN from the scanning session is a reasonable decision in light of the lack of correlation of pN across sessions. An alternative approach would be to use the first-session pN variables to define the groups. As this had to become an exploratory analysis anyway, it would be good to run it both ways to gauge the robustness of the results across two equally reasonable approaches.

This is an intriguing idea, but we disagree that the two approaches are equally reasonable. The logic of the experiment is that the neural data from subjects who are making a relatively high number of swap errors will show a correlate of intrusion from nontargets in the form of a lower orientation recall specificity index. Relabeling the fMRI data according to the screening session could only add noise, because many of the subjects labeled as “low” would, in fact, have been subjects who actually made a “high” number of swap errors during the scanning sessions. Because we already know that the high swap-error subjects had lower orientation recall specificity indices, the effect of relabeling them as “low” in a reanalysis is already known - it would reduce the difference between the two groups. But this would be a trivial consequence of mislabeling a subset of the data, and would have no theoretical value.

- Line 754: Selecting TR by whether the location reconstruction was significant for low-swap subjects biases the correlation in the direction of the hypothesis because it potentially selects a TR in which, simply due to noise, the location specificity of the low-swap subjects (but not the high-swap subjects) was elevated. Therefore, it would be reassuring to see the correlation also reported for the originally planned TR7.

We have done so, and have now added the following: “When we nevertheless carried out the planned Spearman correlation with data from TRs 6 and 7, it was not significant (p > .23).”

- In Figure 7, was the neural signal shifted back in time by the average BOLD delay?

We have now modified the figure legend to read: “Time course of IEM reconstructions of unshifted data from 3O trials.”

- The second sentence of the abstract contains three “versus” statements, making it a bit challenging to unpack what is being compared. I suggest restructuring this for clarity.

We have now rephrased this sentence as follows: “In Experiment 1, fMRI of 16 male and female humans replicated a previous dissociation of the effects of manipulating memory load in comparison to the effects of manipulating context binding, by comparing VWM for one oriented line versus for three lines individuated by their location versus for three “heterogeneous” items drawn from different categories (orientation, color, and luminance):

Additional change. I preparing this revision we realized that the initial submission did not include Table 1 (which contains behavioral results from Experiment 1). It is now included in both the changes-marked version and the clean version of the revised manuscript.
